# Supplementary material for: PADI4-mediated citrullination of histone H3 stimulates HIV-1 transcription
Source: Nat Commun. 2025 Jun 25;16:5393. doi: 10.1038/s41467-025-61029-0 (PMC12198384; doi:10.1038/s41467-025-61029-0)
Supplement: Supplementary file 1 — Supplementary Information [file 41467_2025_61029_MOESM1_ESM.pdf]

Supplementary information

PADI4-mediated citrullination of histone H3 stimulates HIV-1 transcription

Luca Love[1,*], Bianca B Jütte[1,*], Birgitta Lindqvist[1], Hannah Rohdjess[1], Oscar Kieri[2,3], Piotr Nowak[2,3], J Peter Svensson[1,#]

[1] Department of Medicine Huddinge, Center for Infectious Medicine (CIM), Karolinska Institutet, Huddinge, Sweden

[2] Department of Infectious Diseases, Karolinska University Hospital, Stockholm, Sweden

[3] Department of Medicine Huddinge, Division of Infectious Diseases, Karolinska Institutet, Huddinge, Sweden

*These authors contributed equally

[#] Corresponding author, email: peter.svensson@ki.se

Table S1 Clinical characteristics of the study participants

	**viremic (n=14)**	**ART-treated (n=17)**	**P-value***
**Female**	7 (50%)	8 (47%)	1
**Age (years)**	51 (35-58)	53 (49-63)	0.196
**Years on ART**	N/A	12.6 (8.3-15.6)	N/A
**Plasma viremia** (HIV RNA c/ml)	29000 (541-394400)	<50**	**<0.0001**
**CD4+ T-cell count** (10^6/ml)	280 (135-450)	660 (500-770)	**0.0016**
**CD8+ T-cell count** (10^6/ml)	925 (590-1308)	810 (620-980)	0.356
**CD4+/CD8+**	0.25 (0.10-0.43)	0.80 (0.64-1.06)	**0.00013**
**Nadir CD4** (10^6/ml)	170 (56-250)	250 (170-330)	0.146

* the Mann–Whitney U test was applied to compare the continuous variables and Fisher's exact test to analyze the categorical variables. Continuous characteristics are illustrated as median (inter quartile range) and categorical characteristics are illustrated as n (%).

** 15 ART-treated individuals had HIV RNA levels <20 c/ml at the time of study inclusion, 2 ART-treated individuals had HIV RNA level of 27 and 34 c/ml.

Table S2: Enriched gene ontology terms H3cit

		**DMSO_TSS**			**PMAi_TSS**			**DMSO_elongation**			**PMAi_elongation**		
**GO Biological process***	most significant	number of genes	%	corrected p-value	number of genes	%	corrected p-value	number of genes	%	corrected p-value	number of genes	%	corrected p-value
chromatin remodeling	5.3E-10	276	6.6	5.3E-10	244	6.4	4.2E-07	199	6.3	0.000024	221	6.5	3.7E-07
protein phosphorylation	0.000059	92	2.2	0.0039	93	2.4	0.000059						
intracellular signal transduction	0.00027	38	0.9	0.00036	131	3.4	0.00027						
actin cytoskeleton organization	0.00052	79	1.9	0.00052	72	1.9	0.0016						
positive regulation of DNA-templated transcription	0.00076	190	4.5	0.0094	183	4.8	0.00076						

*all GO Biological preocess terms that are found significant (corrected p-value <0.001) in at least one of the four categories.

Table S3: Primer and probe sequences used in this study.

primer/probe name	primer sequence (5´-3´)	5´position in HIV-1 reference genome (HXB2)
Freadth-2 F	GCCCTCAGATGCTRCATATAA	411
Rreadth-1 R	AGAGTCACACAACAGACGG	582
Preadth-1 Probe	/5HEX/TGCCTGTAC/ZEN/TGGGTCTCTCTGGTTAG/3IABkFQ/	444
AflII accessibility F	TGGGAGCTCTCTGGCTAACTA	484
AflII accessibility R	CTGGTTTCCCTTTCGCTTTC	675
5LTR F	GCCTCAATAAAGCTTGCCTTGA	522
5LTR R	GGCGCCACTGCTAGAGATTTT	642
5LTR P728 PROBE	/56-FAM/AAGTAGTGT/ZEN/GTGCCCGTCTG/3IABkFQ/	551
Ψ F	CAGGACTCGGCTTGCTGAAG	692
Ψ R	GCACCCATCTCTCTCCTTCTAGC	797
Ψ Probe	/56-FAM/TTTTGGCGT/ZEN/ACTCACCAGT/3IABkFQ/	758
Tat-Rev mf1 F	CTTAGGCATCTCCTATGGCAGGAA	5956
Tat-Rev mf83 R	GGATCTGTCTCTGTCTCTCTCTCCACC	8459
Mf226mod (Tat-Rev) Probe	/56-FAM/ACCCGACAG/ZEN/GCC/3IABkFQ/	8402
Env 7781 F	AGTGGTGCAGAGAGAAAAAAGAGC	7736
Env 7781 R	GTCTGGCCTGTACCGTCAGC	7851
Env 7781 PROBE	/5HEX/CCTTGGGTT/ZEN/CTTGGGA/3IABkFQ/	7781
Env 7781 hyperm PROBE	/5HEX/CCTTGGGTT/ZEN/CTTGGGA/3IABkFQ/	7781
PADI4 F	CTGTGGTGTTCCAAGACAGC	
PADI4 R	GCTTGGATGTAGCCGATCTC	
PADI4 FAM PROBE	/56-FAM/CCCAACACC/ZEN/CAGCCCCCGCA/3IABkFQ/	
rpp30 3prime F	GTGTGAGTCAATCACTAGACAGAA	
rpp30 3prime R	AAACTGCAACAACATCATAGAGC	
rpp30 3prime PROBE	/56-FAM/AGAGAGCAA/ZEN/CTTCTTCAAGGGCCC/3IABkFQ/	
RPP30 F	GATTTGGACCTGCGAGCG	
RPP30 R	GCGGCTGTCTCCACAAGT	
rpp30 PROBE	/5HEX/CTGACCTGA/ZEN/AGGCTCT/3IABkFQ/	
HBD F	CCCTGGCTCACAAGTACCAT	
HBD R	GCCATACCCTTGAAGTAGGCA	
TNF alpha promoter F	GCCCATGTTGTAGCAAACCCTCAA	
TNF alpha promoter R	AGTAGATGAGGTACAGGCCCTCTGAT	
ACTB F intron spanning	AGCCTCGCCTTTGCCGA	

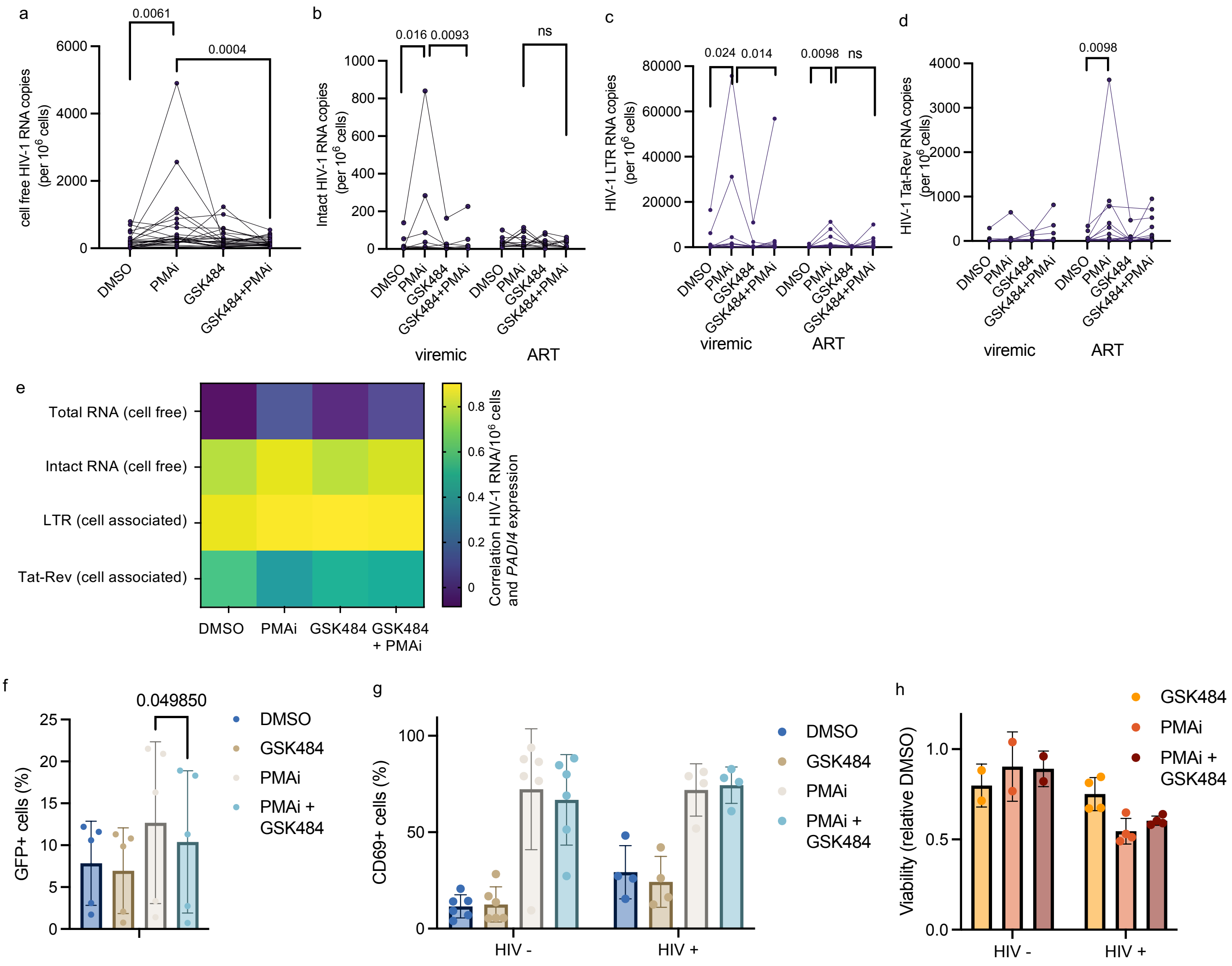

Fig S1: The effect of PADI4 on HIV-1 latency reversal in primary CD4 cells. a Cell free HIV-1 RNA quantification from Fig 1a showing individual data points. **b** Intact HIV-1 RNA copies from Fig 1b showing individual data points. **c** HIV-1 LTR RNA copies from Fig 1d showing individual data points. **d** HIV-1 Tat-Rev RNA copies from Fig 1e showing individual data points. Exact p-values were calculated with two-sided Wilcoxon matched-pairs signed rank test (panels a-d). **e** Correlation between HIV-1 RNA and PADI4 expression. **f** HIV-1 activation level of primary CD4 T cells (n=5) *ex vivo* infected with HIV-1-GFP treated with GSK484. P-values were calculated with multiple paired two-sided T test, correction by Holm-Šídák method. **g** CD69 surface expression level of cells in panel f. **h** Viability of cells in panel f. Data is shown as mean ± SEM. The number of independent experiments is denoted by n. Source data are provided as a Source Data file.

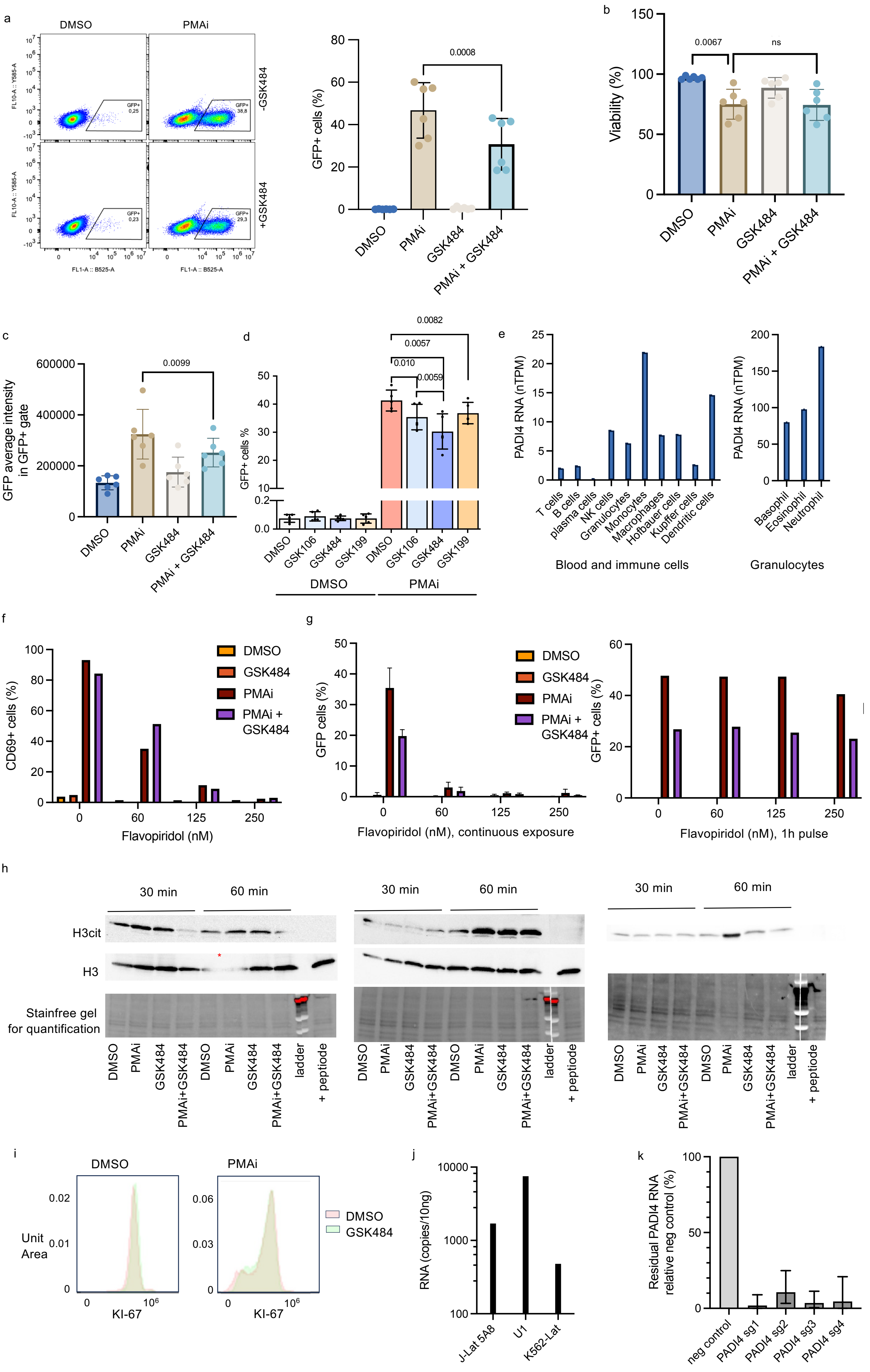
a
DMSO
PMAi
-GSK484
+GSK484
FL10-A :: Y585-A
FL1-A :: B525-A
GFP+ 0,25
GFP+ 38,8
GFP+ 0,23
GFP+ 29,3
GFP+ cells (%)
0.0008
DMSO
PMAi
GSK484
PMAi + GSK484
b
Viability (%)
0.0067
ns
DMSO
PMAi
GSK484
PMAi + GSK484
c
GFP average intensity in GFP+ gate
0.0099
DMSO
PMAi
GSK484
PMAi + GSK484
d
GFP+ cells %
0.0082
0.0057
0.010
0.0059
DMSO
GSK106
GSK484
GSK199
DMSO
GSK106
GSK484
GSK199
DMSO
PMAi
e
PADI4 RNA (nTPM)
T cells
B cells
plasma cells
NK cells
Granulocytes
Monocytes
Macrophages
Hofbauer cells
Kupffer cells
Dendritic cells
Blood and immune cells
PADI4 RNA (nTPM)
Basophil
Eosinophil
Neutrophil
Granulocytes
f
CD69+ cells (%)
DMSO
GSK484
PMAi
PMAi + GSK484
Flavopiridol (nM)
g
GFP cells (%)
DMSO
GSK484
PMAi
PMAi + GSK484
Flavopiridol (nM), continuous exposure
GFP+ cells (%)
Flavopiridol (nM), 1h pulse
h
30 min
60 min
H3cit
H3
Stainfree gel for quantification
DMSO
PMAi
GSK484
PMAi+GSK484
ladder
+ peptide
i
DMSO
PMAi
Unit Area
KI-67
DMSO
GSK484
j
RNA (copies/10ng)
J-Lat 5A8
U1
K562-Lat
k
Residual PADI4 RNA relative neg control (%)
neg control
PADI4 sg1
PADI4 sg2
PADI4 sg3
PADI4 sg4

Fig S2: The effect of PADI4 on HIV-1 latency reversal in cell lines. a Gating for GFP in J-Lat 5A8 cells activated by PMAi and treated with GSK484 (left). Quantification of GFP+ in the same cells (n=6) (right). **b** Viability of activated J-Lat 5A8 cells treated with GSK484. **c** Mean GFP intensity of activated J-Lat 5A8 cells treated with GSK484 (n=6). **d** Activated J-Lat 5A8 cells treated with inactive control compound GSK106 and alternative PADI4 inhibitor GSK199 (n=5). **e** PADI4 expression levels in various cell types. **f** CD69 expression levels in J-Lat 5A8 cells treated with flavopiridol. **g** HIV-1 activation levels in J-Lat 5A8 cells treated with continuous (left) or a 1 h pulse (right) flavopiridol. **h** Replicate immunoblots using antibodies against H3cit and H3 in J-Lat 5A8 cells. **i** Ki-67 expression in unstimulated (DMSO, left) and activated (PMAi, right) J-Lat 5A8 cells, treated with GSK484 (green) or vehicle control DMSO (pink). **j** *PADI4* expression in cell lines used in this study relative to *RPP30*, quantified by ddPCR. **k** *PADI4* expression quantified by ddPCR in CRISPRi K562-Lat using targeting *PADI4* compared to non-targeting negative control. Data is normalized to the non-targeting negative control sample. Error bars represent the 95% confidence interval.

Data is shown as mean $\pm$ SEM for panels a-d, f-h. The number of independent experiments is denoted by n. Exact p-values were calculated with two-sided unpaired Students t tests. Source data are provided as a Source Data file.

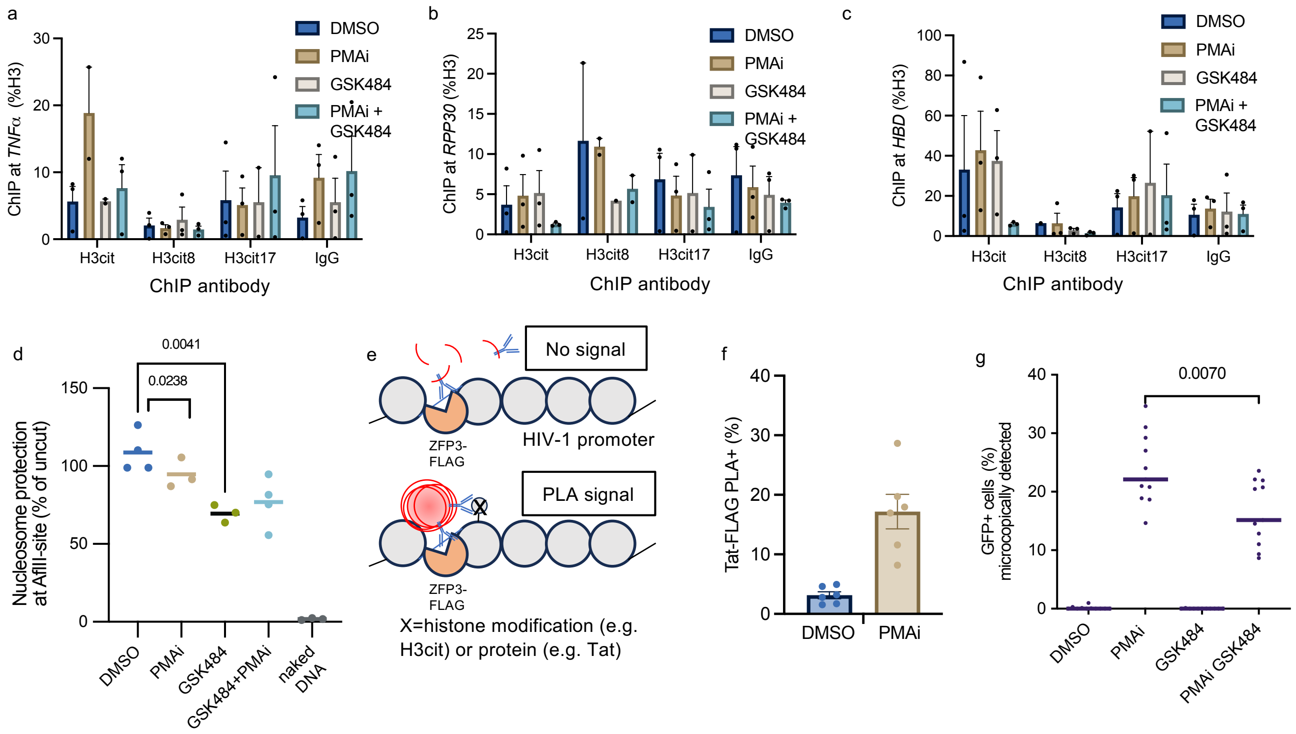

Fig S3: Chromatin measurements

a ChIP quantification of H3cit residues and IgG at the *TNFα* promoter, normalized to H3 (n=3). **b** ChIP quantification of H3cit residues and IgG at *RPP30*, normalized to H3 (n=3). **c** ChIP quantification of H3cit residues and IgG at *HBD*, normalized to H3 (n=3). **d** *AflII*-site protection assay in activated J-Lat 5A8 cells treated with GSK484 (n=4). **e** Schematic of the PLA-ZFP experiment. **f** Control PLA experiments with antibodies against FLAG and Tat in 1C10 cells exposed to DMSO (negative control) and PMAi (positive control) for 24 h (n=6). **g** Microscopy quantification of HIV-1 activation levels (GFP) in J-Lat 5A8 cells treated with GSK484 (n=11). Data is shown as mean ± SEM for panels a-d, f-h. The number of independent experiments is denoted by n. Exact p-values were calculated with two-sided unpaired Students t tests. Source data are provided as a Source Data file.

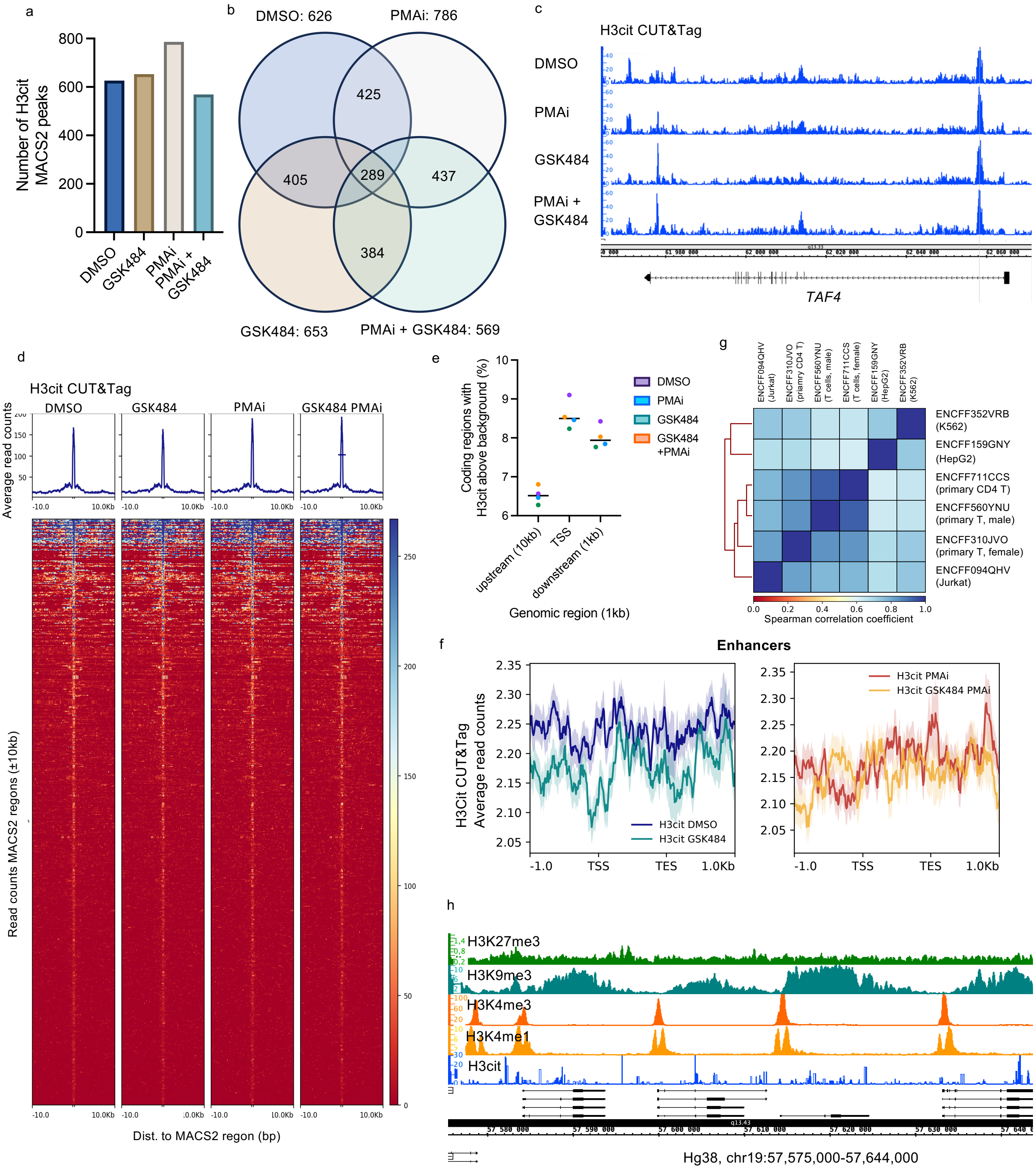

i

	non-I.S.	latent	productive	reactivatable
non-I.S.	-	0.00012	1.60E-14	0.027
latent		-	0.00075	0.4
productive			-	0.61
reactivatable				-

Fig S4: Genome-wide H3cit CUT&Tag results. a Number of MACS2 H3cit peaks in activated J-Lat 5A8 cells treated with GSK484. **b** Overlap of peaks identified from Fig S4a in the different conditions. **c** H3cit peaks in the *TAF4* gene across the different conditions.

Fig S4: Genome-wide H3cit CUT&Tag results (continued). d Metagene plot and heatmap for H3cit peaks in DMSO, GSK484, PMAi and PMAi/GSK484 treated conditions in 5A8 cells. **e** Relative enrichment of H3cit over background in 1kb genomic regions covering the Transcription Start Site (TSS), 10kb upstream, and 10kb downstream of the TSS. **f** Metagene plots of the H3cit levels at enhancer regions. **g** Correlation of H3K4me3 ChIP-seq profiles from the ENCODE data base. The average read counts over the transcript regions were used to generate the plot. **h** ChIP-seq or CUT&Tag profiles of five chromatin marks in a genomic region at *chr19*. **i** P-values (two-way unpaired Students t test) when comparing H3cit levels at sites of proviral integration leading to latent, productive or reactivatable HIV-1 infection. Non-I.S. denotes random genomic sites not associated with HIV-1 integration in this data set. Source data are provided as a Source Data file.

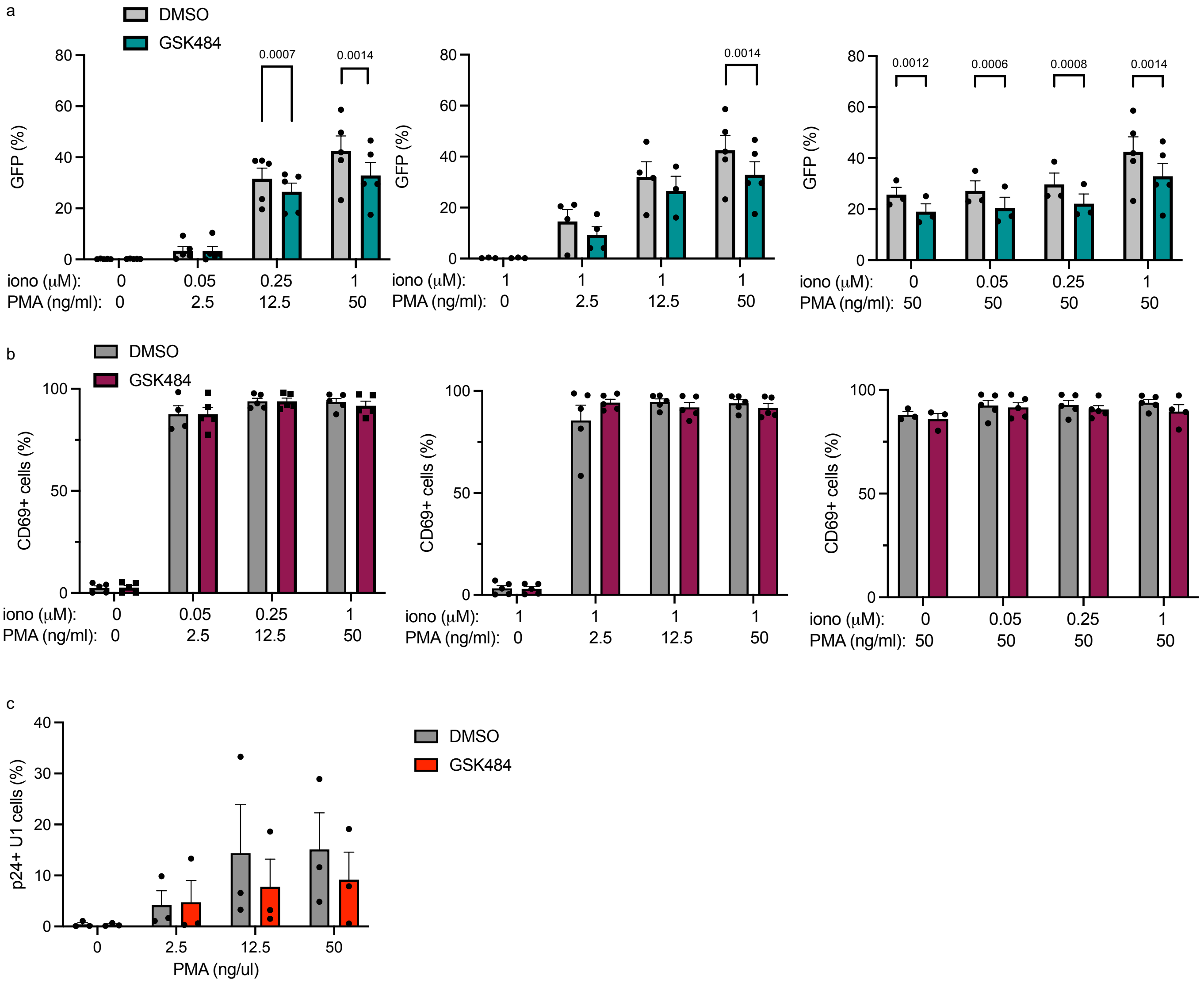

Fig S5: Dose-response of PMA and ionomycin in combination with PADI4-inhibitor GSK484). **a** HIV latency reversal as measured by GFP in J-Lat 5A8 cells exposed to increasing doses of PMA and/or ionomycin (n=5). **b** Cellular activation measured by surface marker CD69 in J-Lat 5A8 cells exposed to increasing doses of PMA and ionomycin (n=5). **c** HIV latency reversal as measured by intracellular p24 in U1 ecells exposed to increasing dose of PMA (n=3).

Data is shown as mean ± SEM. The number of independent experiments is denoted by n. Exact p-values were calculated with two-sided paired Students t tests. Source data are provided as a Source Data file.

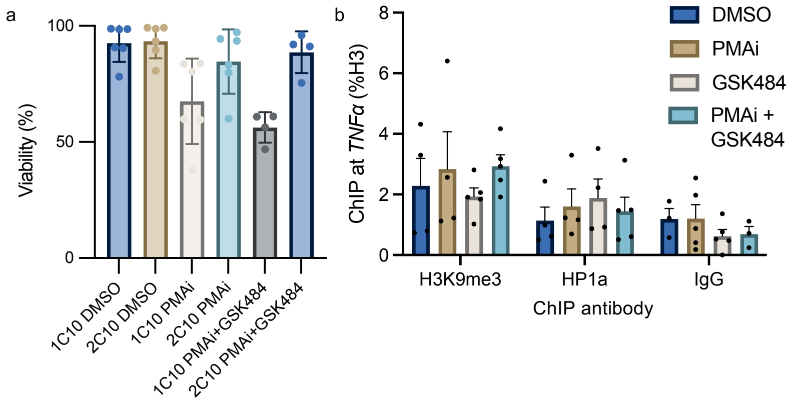

Fig S6: Heterochromatin effects. a Cell viability in activated J-Lat 1C10 and J-Lat 2C10 treated with GSK484 (n=6). **b** H3K9me3 and HP1α levels at *TNFα*, quantified by ChIP (n=5). Source data are provided as a Source Data file.

Gating strategy

1	2	3	4	5
Lymphocytes	Singlets	Viability (LIVE/DEAD™ Fixable Violet)<br>OR Alive (Zombie NIR™ Fixable)	Anti-CD25, Anti-CD69	GFP + cells
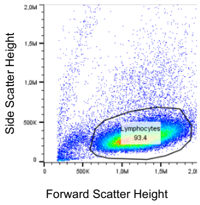	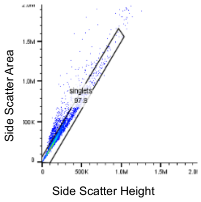	PMAi treated cells<br>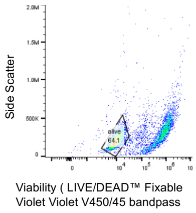<br>PMAi treated cells<br>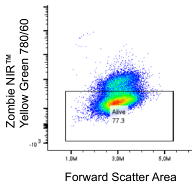	DMSO treated cells<br>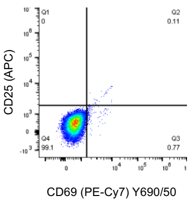<br>PMAi treated cells<br>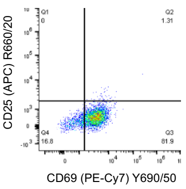	DMSO treated cells<br>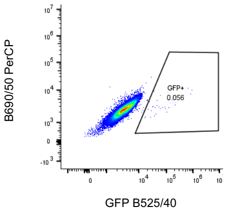<br>PMAi treated cells<br>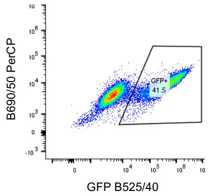

Fig S7: Gating for analysis of flow cytometry data.
