# Supplementary material for: PADI4-mediated citrullination of histone H3 stimulates HIV-1 transcription
Source: Nat Commun. 2025 Jun 25;16:5393. doi: 10.1038/s41467-025-61029-0 (PMC12198384; doi:10.1038/s41467-025-61029-0)
Supplement: Supplementary file 2 — Description Of Additional Supplementary File [file 41467_2025_61029_MOESM2_ESM.pdf]

Description of additional supplementary files

Supplementary data 1.

Description: H3cit CUT&Tag data over genes

Supplementary data 2.

Description: ChIP-PCR Ct values
