# Supplementary material for: PADI4-mediated citrullination of histone H3 stimulates HIV-1 transcription
Source: Nat Commun. 2025 Jun 25;16:5393. doi: 10.1038/s41467-025-61029-0 (PMC12198384; doi:10.1038/s41467-025-61029-0)
Supplement: Supplementary file 5 — Reporting Summary [file 41467_2025_61029_MOESM5_ESM.pdf]

nature portfolio

Corresponding author(s): Peter Svensson

Last updated by author(s): Jun 8, 2025

Reporting Summary

Nature Portfolio wishes to improve the reproducibility of the work that we publish. This form provides structure for consistency and transparency in reporting. For further information on Nature Portfolio policies, see our Editorial Policies and the Editorial Policy Checklist.

Statistics

For all statistical analyses, confirm that the following items are present in the figure legend, table legend, main text, or Methods section.

n/a	Confirmed	
☐	☒	The exact sample size (n) for each experimental group/condition, given as a discrete number and unit of measurement
☐	☒	A statement on whether measurements were taken from distinct samples or whether the same sample was measured repeatedly
☐	☒	The statistical test(s) used AND whether they are one- or two-sided<br>*Only common tests should be described solely by name; describe more complex techniques in the Methods section.*
☐	☒	A description of all covariates tested
☐	☒	A description of any assumptions or corrections, such as tests of normality and adjustment for multiple comparisons
☐	☒	A full description of the statistical parameters including central tendency (e.g. means) or other basic estimates (e.g. regression coefficient) AND variation (e.g. standard deviation) or associated estimates of uncertainty (e.g. confidence intervals)
☐	☒	For null hypothesis testing, the test statistic (e.g. F, t, r) with confidence intervals, effect sizes, degrees of freedom and P value noted<br>*Give P values as exact values whenever suitable.*
☒	☐	For Bayesian analysis, information on the choice of priors and Markov chain Monte Carlo settings
☒	☐	For hierarchical and complex designs, identification of the appropriate level for tests and full reporting of outcomes
☒	☐	Estimates of effect sizes (e.g. Cohen's d, Pearson's r), indicating how they were calculated

Our web collection on statistics for biologists contains articles on many of the points above.

Software and code

Policy information about availability of computer code

Data collection	No software was used
Data analysis	Graphpad prism (version 10.3), Bowtie2 (version 2.5.2) and Galaxy (version 24.1.2) were used to analyze this study.

For manuscripts utilizing custom algorithms or software that are central to the research but not yet described in published literature, software must be made available to editors and reviewers. We strongly encourage code deposition in a community repository (e.g. GitHub). See the Nature Portfolio guidelines for submitting code & software for further information.

Data

Policy information about availability of data

All manuscripts must include a data availability statement. This statement should provide the following information, where applicable:

- Accession codes, unique identifiers, or web links for publicly available datasets
- A description of any restrictions on data availability
- For clinical datasets or third party data, please ensure that the statement adheres to our policy

The datasets generated in this study are publicly available through the Gene Expression Omnibus (GEO) GSE273899.

Research involving human participants, their data, or biological material

Policy information about studies with human participants or human data. See also policy information about sex, gender (identity/presentation), and sexual orientation and race, ethnicity and racism.

Reporting on sex and gender	No sex or gender based analysis was performed. Sex of study participants is recorded in supplementary table 1.
Reporting on race, ethnicity, or other socially relevant groupings	No reporting on race, ethnicity or other socially relevant groupings.
Population characteristics	Age, HIV-subtype, HIV virus levels and ART/no ART reported.
Recruitment	Study participants were recruited from the HIV unit at Department of Infectious Diseases, Karolinska University Hospital. Between April 2021 and November 2022, 31 study participants were included in this study.
Ethics oversight	This study was approved by the Regional Ethics Committee (Regionala Etikprövningsnämnden Stockholm, Reg#2018/102-31)

Note that full information on the approval of the study protocol must also be provided in the manuscript.

Field-specific reporting

Please select the one below that is the best fit for your research. If you are not sure, read the appropriate sections before making your selection.

☒ Life sciences ☐ Behavioural & social sciences ☐ Ecological, evolutionary & environmental sciences

For a reference copy of the document with all sections, see nature.com/documents/nr-reporting-summary-flat.pdf

Life sciences study design

All studies must disclose on these points even when the disclosure is negative.

Sample size	Sample size was chosen by estimation of the effect and thereby statistical calculations to achieve power.
Data exclusions	Data were excluded when technical failure occured. For the ddPCR, primers are not able to universally bind to sequences of all individuals. We have stated in the text how many fell point fell out of analysis.
Replication	For many of the samples, replicate measurements were performed.
Randomization	Covariates were controlled by matching groups of age and HIV-1 subtype.
Blinding	Blinding was not relevant to this study as it is exploratory.

Reporting for specific materials, systems and methods

We require information from authors about some types of materials, experimental systems and methods used in many studies. Here, indicate whether each material, system or method listed is relevant to your study. If you are not sure if a list item applies to your research, read the appropriate section before selecting a response.

Materials & experimental systems

n/a	Involved in the study	
☐	☒	Antibodies
☐	☒	Eukaryotic cell lines
☒	☐	Palaeontology and archaeology
☒	☐	Animals and other organisms
☐	☒	Clinical data
☒	☐	Dual use research of concern
☒	☐	Plants

Methods

n/a	Involved in the study	
☐	☒	ChIP-seq
☐	☒	Flow cytometry
☒	☐	MRI-based neuroimaging

Antibodies

Antibodies used	Anti-Histone H3 (citrulline R2) antibody [EPR17703] Abcam Cat# ab176843<br>Anti-Histone H3 (citrulline R8) antibody [EPR20358-13] Abcam Cat# ab219406<br>Anti-Histone H3 (citrulline R17) antibody [EPR20358-120] Abcam Cat# ab219407

	Human Histone H3 (citrulline R2 + R8 + R17) Abcam Cat# 32876<br>Anti-Histone H3 antibody - Nuclear Loading Control and ChIP Grade (ab1791) Abcam Cat# ab1791<br>Anti HIV-1 Tat Antibody ChIP-Grade Abcam Cat# ab43014<br>FLAG Tag Polyclonal Antibody Bioss Antibodies Cat# BS-0965R<br>CD25 Mouse anti Human, APC, Clone: m A251 BD Biosciences Cat# 555434<br>Anti-CD25 Mouse Monoclonal Antibody (PE (Phycoerythrin)) [clone: BC96] Biolegend Cat#302605<br>APC Mouse Anti-Human CD69 Clone FN50 BD Biosciences Cat# 560967<br>PE-Cy7 Mouse Anti-Human CD69 Clone FN50 BD Biosciences Cat# 561928, Lot#9136867<br>APC anti-human Ki-67 Antibody Biolegend Cat# 350513<br>FITC-conjugated anti-GFP Abcam Cat# ab6662
Validation	Data validated in controls of this study.

Eukaryotic cell lines

Policy information about cell lines and Sex and Gender in Research

Cell line source(s)	5A8 cells were obtained from the lab of Eric Verdin.
Authentication	Sequencing of HIV-1 insert
Mycoplasma contamination	All cell lines tested negative for mycoplasma contamination.
Commonly misidentified lines (See ICLAC register)	No comminly misidentified cell lines used.

Clinical data

Policy information about clinical studies

All manuscripts should comply with the ICMJE guidelines for publication of clinical research and a completed CONSORT checklist must be included with all submissions.

Clinical trial registration	This study was approved by the Regional Ethics Committee (Regionala Etikprövningsnämnden Stockholm, Reg#2018/102-31).
Study protocol	written informed consent was obtained from all subjects. All methods of this study was performed in accordance with the principles outlined in the Declaration of Helsinki. Inclusion criteria were either a) on ART and with suppressed viremia for at least 6 years (plasma viral load <50 copies/ml) b) not on ART and with viremia (plasma viral load >50 copies/ml). The current study complies with STROBE guidelines.
Data collection	Study participants were recruited from the HIV unit at Department of Infectious Diseases, Karolinska University Hospital. Between April 2021 and October 2021, 31 study participants were included in this study.
Outcomes	Primary outcome was viral RNA/DNA in cells/supernatant after ex vivo treatment. The outcome was assessed by ddPCR. The data were analyzed anonymously.

Plants

Seed stocks	*Report on the source of all seed stocks or other plant material used. If applicable, state the seed stock centre and catalogue number. If plant specimens were collected from the field, describe the collection location, date and sampling procedures.*
Novel plant genotypes	*Describe the methods by which all novel plant genotypes were produced. This includes those generated by transgenic approaches, gene editing, chemical/radiation-based mutagenesis and hybridization. For transgenic lines, describe the transformation method, the number of independent lines analyzed and the generation upon which experiments were performed. For gene-edited lines, describe the editor used, the endogenous sequence targeted for editing, the targeting guide RNA sequence (if applicable) and how the editor was applied.*
Authentication	*Describe any authentication procedures for each seed stock used or novel genotype generated. Describe any experiments used to assess the effect of a mutation and, where applicable, how potential secondary effects (e.g. second site T-DNA insertions, mosiacism, off-target gene editing) were examined.*

ChIP-seq

Data deposition

☒ Confirm that both raw and final processed data have been deposited in a public database such as GEO.

☒ Confirm that you have deposited or provided access to graph files (e.g. BED files) for the called peaks.

Data access links<br>*May remain private before publication.*	The datasets generated in this study is publicly available through the Gene Expression Omnibus (GEO) GSE273899.
Files in database submission	DMSO_5A8_H3cit_rep_1_S1_R1_001.fastq.gz

Files in database submission	DMSO_5A8_H3cit_rep_1_S1_R2_001.fastq.gz<br>DMSO_5A8_H3cit_rep_2_S5_R1_001.fastq.gz<br>DMSO_5A8_H3cit_rep_2_S5_R2_001.fastq.gz<br>DMSO_5A8_H3cit_rep_3_S9_R1_001.fastq.gz<br>DMSO_5A8_H3cit_rep_3_S9_R2_001.fastq.gz<br>GSK484_5A8_H3cit_rep_2_S6_R1_001.fastq.gz<br>GSK484_5A8_H3cit_rep_2_S6_R2_001.fastq.gz<br>GSK484_5A8_H3cit_rep_3_S10_R1_001.fastq.gz<br>GSK484_5A8_H3cit_rep_3_S10_R2_001.fastq.gz<br>GSK484_5A8_H3cit_rep1_S2_R1_001.fastq.gz<br>GSK484_5A8_H3cit_rep1_S2_R2_001.fastq.gz<br>PMAi_5A8_H3cit_rep_2_S7_R1_001.fastq.gz<br>PMAi_5A8_H3cit_rep_2_S7_R2_001.fastq.gz<br>PMAi_5A8_H3cit_rep_3_S11_R1_001.fastq.gz<br>PMAi_5A8_H3cit_rep_3_S11_R2_001.fastq.gz<br>PMAi_5A8_H3cit_rep1_S3_R1_001.fastq.gz<br>PMAi_5A8_H3cit_rep1_S3_R2_001.fastq.gz<br>PMAi_GSK484_5A8_H3cit_rep_2_S8_R1_001.fastq.gz<br>PMAi_GSK484_5A8_H3cit_rep_2_S8_R2_001.fastq.gz<br>PMAi_GSK484_5A8_H3cit_rep_3_S12_R1_001.fastq.gz<br>PMAi_GSK484_5A8_H3cit_rep_3_S12_R2_001.fastq.gz<br>PMAi_GSK484_5A8_H3cit_rep1_S4_R1_001.fastq.gz<br>PMAi_GSK484_5A8_H3cit_rep1_S4_R2_001.fastq.gz<br>H3cit_PMAi.bigwig<br>H3cit_DMSO.bigwig<br>H3cit_GSK484_PMAi.bigwig<br>H3cit_GSK484.bigwig
Genome browser session (e.g. UCSC)	https://www.ncbi.nlm.nih.gov/gds/?term=GSE273899[Accession]

Methodology

Replicates	Three replicates were performed on separate days for all data points.
Sequencing depth	Between 15 and 44 million reads per sample resulting in a range of 0.6-1.8X total genome coverage. Paired-end reads.
Antibodies	Human Histone H3 (citrulline R2 + R8 + R17) Abcam Cat# 32876
Peak calling parameters	macs2 callpeak -t --name filename --format BAMPE --gsize '2700000000' --keep-dup '1' --d-min 20 --buffer-size 100000 --qvalue '0.05' --mfold '5' '50' --bw '300'
Data quality	All samples passed quality check based on read quality and quantity; mappability and clustering within replicates.
Software	Bowtie2 (2.5.2), Galaxy (version 24.1.2).

Flow Cytometry

Plots

Confirm that:

☒ The axis labels state the marker and fluorochrome used (e.g. CD4-FITC).

☒ The axis scales are clearly visible. Include numbers along axes only for bottom left plot of group (a 'group' is an analysis of identical markers).

☒ All plots are contour plots with outliers or pseudocolor plots.

☒ A numerical value for number of cells or percentage (with statistics) is provided.

Methodology

Sample preparation

Cells were stained with LIVE/DEAD Fixable Violet Dead Cell Stain (Thermo Scientific, Cat# L34955) or Zombie NIR™ Fixable Viability Kit (Biolegend Cat# 423105) and fixed in 2% formaldehyde for 15 min.

When staining for CD25 and CD69. Cells were washed with PBS (Gibco #18912014) + 0.5% BSA (Gibco Cat# 15260037 (FACS buffer) then incubated with 1/100 CD25 (BD, Cat#555434) and CD69 1/20 (BD, Cat# 560711) antibody diluted in FACS Buffer for 30 min at 4°C. Cells were then washed with 100ul of FACS buffer then resuspended in 100ul FACS buffer and 1/1000 dilution of Live/dead stain (Thermo Scientific, Cat# L34964) and incubated for 15 min at 4°C. Cells were washed in 100 µl FACS buffer then resuspended in 2% formaldehyde PBS for 15 min in the dark at room temperature. Finally, cells were resuspended in 100 µl of ice-cold PBS prior to flow cytometry.

When staining for KI-67 cells were washed twice with PBS and resuspended by vortexing at low speed while adding ice cold 70% Ethanol. This was followed by a 1 h incubation at -20°C, three washed with FACS buffer then resuspension in FACS buffer

at 1x106 cells/ml. 5 µl of APC anti-human Ki-67 Antibody (BioLegend, CAT# 350513) in 100 µL of cell solution. This was incubated for 30 min in the dark at room temperature, washed with FACS buffer then resuspended in 100 µl of ice-cold PBS prior to flow cytometry.

Instrument	CytoFLEX S (Beckman Coulter)
Software	Flowjo 10.1
Cell population abundance	% GFP+ cells after all gating procedures ranged from 0.01 to 45% depending on the treatment
Gating strategy	Individual flow droplets were gated for lymphocytes, Singlets, viability, then GFP and CD25/CD69 or KI-67

☒ Tick this box to confirm that a figure exemplifying the gating strategy is provided in the Supplementary Information.
